# The Emerging Roles of Circ-ABCB10 in Cancer

**DOI:** 10.3389/fcell.2022.782938

**Published:** 2022-05-13

**Authors:** Zhenjun Huang, Renfeng Shan, Wu Wen, Jianfeng Li, Xiaohong Zeng, Renhua Wan

**Affiliations:** ^1^ Department of General Surgery, The First Affiliated Hospital of Nanchang University, Nanchang University, Nanchang, China; ^2^ Imaging Department, The First Affiliated Hospital of Nanchang University, Nanchang University, Nanchang, China

**Keywords:** circRNA, circ-ABCB10, cancer, microRNA, tumor biology

## Abstract

Circular RNAs (circRNAs) are non-coding RNAs (ncRNAs) without 5′ caps and 3′ tails, which are formed from precursor mRNAs (pre-mRNAs) that are inversely back-spliced by exons. CircRNAs are characterized by a covalently closed circular structure and are abundantly expressed in eukaryotic cells. With the development of RNA-sequencing, it was discovered that circRNAs play important roles in the regulation of numerous human genes and are related to the occurrence, development, and prognosis of diseases. Studies in various cancers have revealed that circRNAs have both positive and negative effects on the occurrence and development of tumors. Circ-ABCB10, a circular RNA originating from exons of ABCB10 located on chromosome 1q42, has been proven to play an important role in different types of cancers. Here, we report the primary findings of recent research studies by many contributors about the roles of circ-ABCB10 in cancer and clearly formulate its influence and functions in different aspects of cancer biology, which gives us a broad picture of circ-ABCB10. Thus, this study aimed to generalize the roles of circ-ABCB10 in the diagnosis and treatment of different types of tumors and its related miRNA genes. In this way, we wish to provide a sufficient understanding and assess the future development direction of the research on circ-ABCB10.

## Introduction

Circular RNAs (circRNAs) are a class of endogenous single-stranded closed circular RNAs formed by reverse splicing and covalent binding without a 5′ cap and 3′ poly (a) tail (Sanger et al., 1976; Chen, 2016; Pamudurti et al., 2017; Kristensen et al., 2018; Qu et al., 2018). CircRNAs were first discovered in RNA viruses in 1976 (Sanger et al., 1976) and were initially considered “splicing noise” in organisms but have become a research hotspot in the meantime (Kristensen et al., 2018). With the rapid development of RNA-sequencing technology, many circRNAs have been discovered. To date, more than 30,000 cyclic RNAs with unique structures have been identified and have attracted increasing attention. Most circRNAs are evolutionarily conserved across species (Pamudurti et al., 2017). CircRNAs can originate from introns, exons, or from both introns and exons (Su et al., 2019; Yu and Kuo, 2019). Because circRNAs have a specific closed-ring structure, they are more resistant to exonucleases than linear RNAs (Salzman et al., 2013; Shang et al., 2019). In addition, most circRNAs are usually found in the cytoplasm and are derived from protein-coding genes. These genes contain one or more exons toward the 5′ cap of the gene, with long introns on both sides (Dong et al., 2017a; Nicolet et al., 2018). The long introns containing the wing region will become circRNAs, which usually contain specific sequences (Qu et al., 2015). They can induce the formation of circRNAs by mutually complementing with circRNA promoters and are usually expressed in a cell type– or tissue-specific manner (Patop and Kadener, 2018; Shi et al., 2020). Moreover, it was found that circRNAs are abnormally expressed in colorectal cancer (CRC) and osteoarthritis (Bachmayr-Heyda et al., 2015; Yang et al., 2020a). Thus, circRNAs are widely expressed in various human cell types and will perhaps become a new direction in research on disease biomarkers for aging (Bahn et al., 2015) and therapy (Hou and Zhang, 2017; Tang et al., 2017). According to gene structures and their specific molecular cycling mechanisms, circRNAs are divided into four types: exonic circRNAs (ecRNAs) (Salzman et al., 2012), circular intronic RNAs (ciRNAs) (Zhang et al., 2013), exon–intron circRNAs (eIciRNAs) (Li et al., 2015), and intergenic circRNAs (Tang and Hann, 2020). Generally speaking, ecRNAs are mainly found in the cytoplasm (Memczak et al., 2013) and regulate the expression of genes. CiRNAs and eIciRNAs tend to be localized in the nucleus and play a significant role in regulating parental genes.

Studies have shown that circRNAs produced *via* back-splicing feature different biogenesis than typical splicing of linear RNA. First, acting as miRNA sponges, circRNAs are more likely to bind to other miRNAs and are known as “super sponges” (Hansen et al., 2013). According to previous reports, circRNAs can inhibit miRNAs from binding to their target genes (Thomas and Sætrom, 2014). Second, by binding to proteins and RNA-binding proteins (RBPs), circRNAs can affect their function and interaction with other proteins (Zang et al., 2020). Studies demonstrate that RBPs can also regulate the formation of circRNAs by forming RNA–protein complexes (RPCs) (Conn et al., 2015). Third, circRNAs retained in the nucleus can regulate alternative splicing, transcription, or translation (Li et al., 2018a; Guarnerio et al., 2019). For example, Circ-ubr5 might undergo a specific RNA–RNA interaction by binding to the splicing regulator QKI(Qin et al., 2018). CircITGA7 could increase the transcriptional expression of integrin alpha 7 (ITGA7) by inhibiting RAS-responsive element-binding protein 1 (RREB1) (Li et al., 2018a).

Moreover, circRNAs can function as autophagy regulators, affecting tumorigenesis. For example, Circ_104075 was found to act as an autophagy regulator in glioma cells (Chi et al., 2019). In general, the abnormal expression of circRNAs is associated with the occurrence and progression of human cancer by affecting the growth, migration, invasion, proliferation, and other pathological processes of cells (Conn et al., 2015; Khan et al., 2016; Dong et al., 2019). In addition, circRNAs are also associated with clinicopathological characteristics, such as lymph node metastasis, differentiation, or distant metastasis. All of these findings provide the basis for potential biomarkers and therapeutic targets in the diagnosis and treatment of human cancers. In addition, circRNAs can act as a protein sponge to adsorb one or more proteins through binding sites, thus acting as a protein scaffold to directly mediate protein–protein interactions and regulate gene expression. Recent studies have found that the abnormal expression of Circ-ABCB10, also known as hsa_circ_000871, may be involved in the occurrence and development of many different tumors, such as esophageal squamous carcinoma cells, glioma, non–small cell lung cancer, oral squamous cell carcinoma, lung cancer, epithelial ovarian cancer, breast cancer, thyroid cancer, and hepatocellular carcinoma (Cortés-López and Miura, 2016; Wang et al., 2017). Thus, it is significant to summarize the function of Circ-ABCB10 in the occurrence and progression of human cancers.

## The Features and Biological Functions of Circ-ABCB10

**FIGURE 1:**
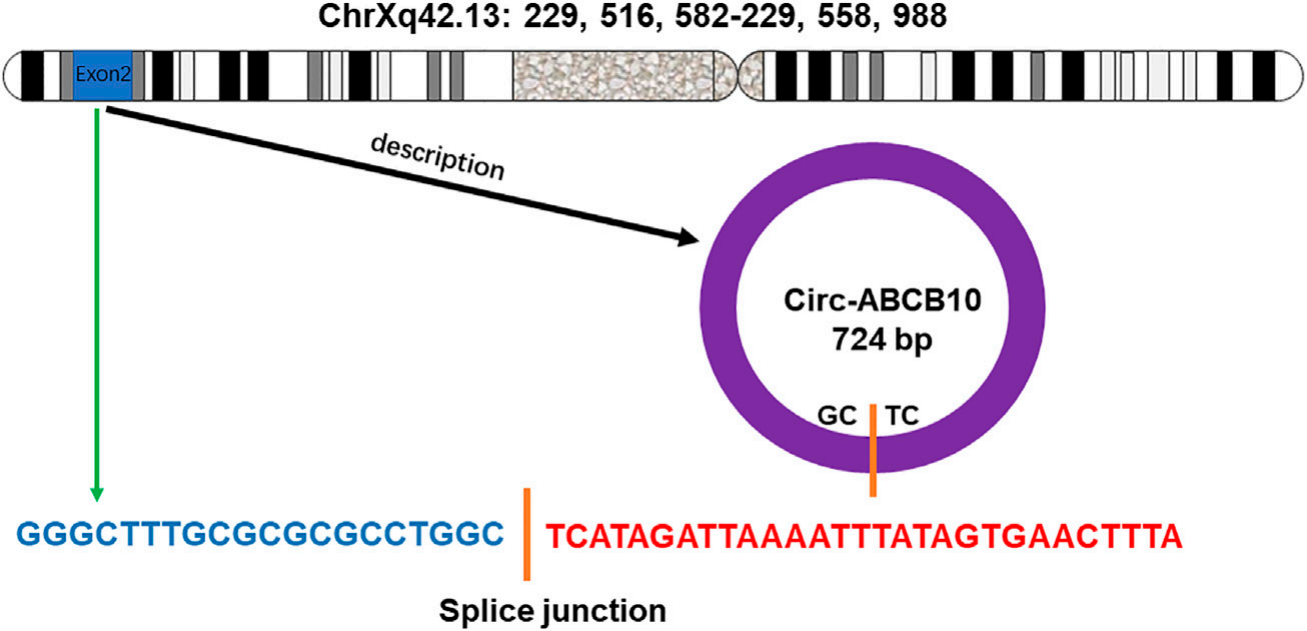
Gene encoding circ-ABCB10 is located on the chromosome Xq42.13. The antisense strand of circ-ABCB10 undergoes back-splicing of the 5′ and 3′ ends to form circular RNA. The green arrow indicates the “head-to-tail” splicing site of circ-ABCB10.

Circ-ABCB10 originates from exons 2 and 3 of the ABCB10 gene, located on chromosome 1 (Duan et al., 2020). The antisense strand of Circ-ABCB10 undergoes back-splicing of the 5′ and 3′ ends to form circular RNA (Figure 1) With specific expression in different developmental stages and tissues, Circ-ABCB10 was first reported to promote breast cancer proliferation and migration by sponging miR-1271 (Chen et al., 2019). It was also found that Circ-ABCB10 is highly expressed in human brain regions such as the forebrain, cerebellum, occipital lobe, frontal cortex, and parietal lobe (Memczak et al., 2013). Different from linear RNAs, circRNAs are single-chain circular RNAs without 5ʹ to 3ʹ polarity or a polyadenylated tail. This blocked structure makes circ-ABCB10 more resistant to RNA degradation. Due to these unique characteristics, circ-ABCB10 is related to several characteristics of cancers (Jayson et al., 2014). Some studies have demonstrated that circ-ABCB10 could stimulate tumor growth (Liang et al., 2017; Zhao et al., 2021) and insulin resistance (IR) (Lux and Bullinger, 2018; Ouyang et al., 2018). Furthermore, the high expression of circ-ABCB10 was closely related to the pathological grade and tumor lymph node metastasis stage (Luo et al., 2018). Thus, circ-ABCB10 may be a promising diagnostic and prognostic biomarker and a target for novel treatment strategies in the future.

## Circ-ABCB10 Functions as a miRNA Sponge

**FIGURE 2:**
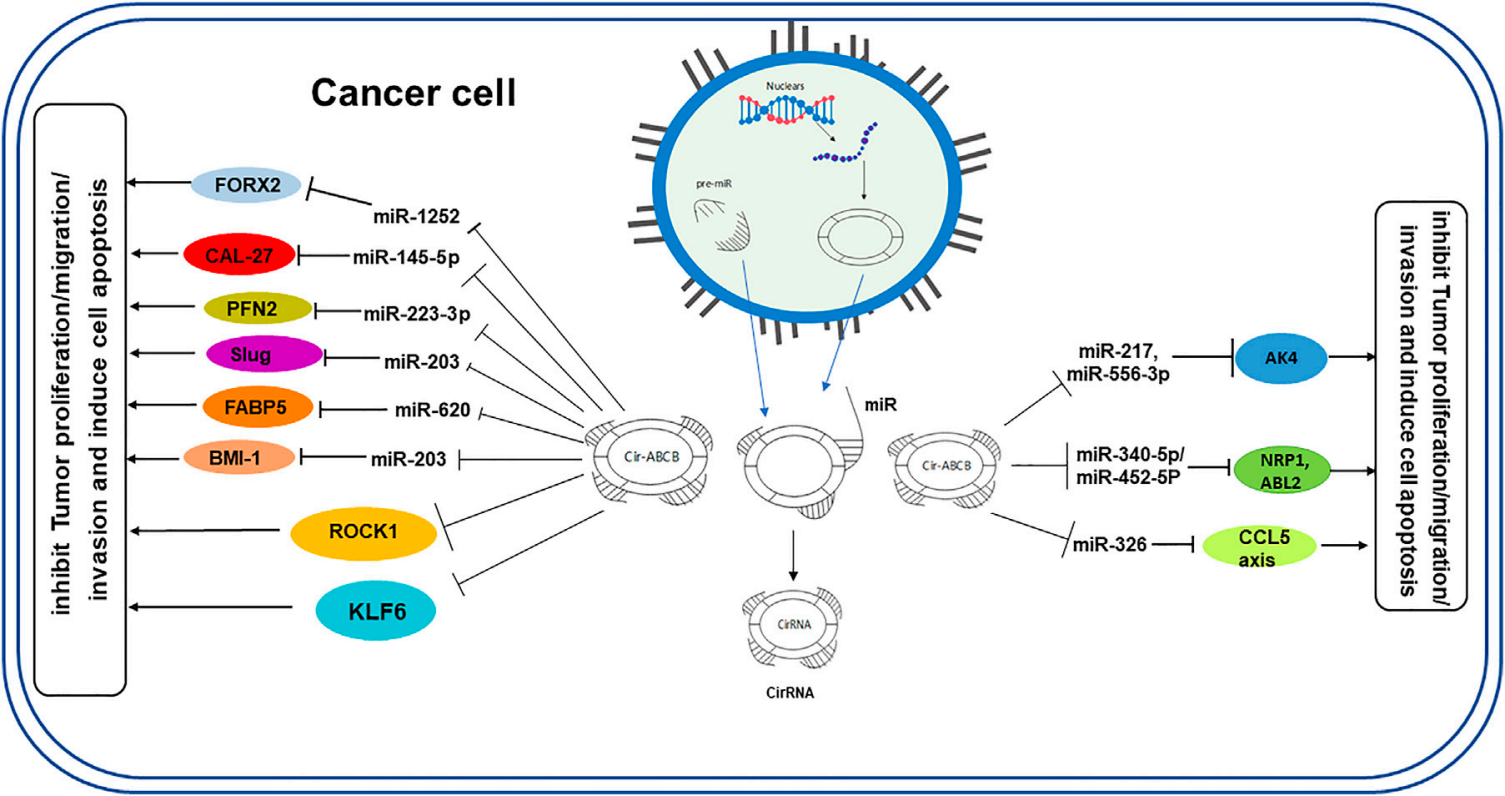
Overview of circ-ABCB10 function as a miRNA sponge in the proliferation and metastasis of various cancer cell types. Circ-ABCB10 is released from the nucleus, acting as a sponge of miRNAs that regulate target genes to promote or inhibit tumor growth.

Like lncRNAs, the main role of circRNAs in molecular regulation is to act as a “sponge” to absorb functional miRNA to decrease their abundance in the cytoplasm and thereby regulate gene expression (Dong et al., 2017b; Li et al., 2017). Among the target miRNAs that can be regulated by circRNAs, it was demonstrated that miR-1252 can provide potential biomarkers and therapeutic targets in non–small cell lung cancer (Tian et al., 2019) and epithelial ovarian cancer (Chen et al., 2019). Similarly, it was also found that circ-ABCB10 can sponge miR-1271, which may have a complementary sequence, possibly regulating cell proliferation and migration of breast cancer and epithelial ovarian cancer cells (Liang et al., 2017; Lin et al., 2021; Zhao et al., 2021). Furthermore, since conserved miR-1271 target sites on circ-ABCB10 are complementary to miR-1271, these sites could be a lodging site for transport (Lin et al., 2021). In addition, circ-ABCB10 was reported to sponge miR-145-5p, affecting the miR-620/FABP5 axis, miR-1252 FOXR2, and miR-670-3p in oral squamous cell carcinoma, glioma, non–small cell lung cancer, and esophageal squamous cell carcinoma, respectively (Tian et al., 2019; Chen et al., 2020; Sun et al., 2020; Zhang et al., 2020). Furthermore, circ-ABCB10 was also found to promote glycolysis and colony formation by sponging miR-229-3p (Zhao et al., 2020). In addition, Circ-ABCB10 can promote angiogenesis *via* the microRNA-29b-3p/vascular endothelial growth factor A-axis (Tang et al., 2020) (Figure 2).

## The Roles of Circ-ABCB10 in Cancer Progression

With the progress of circRNA research, relevant studies demonstrated that Circ-ABCB10 is abnormally expressed in various cancers, such as esophageal squamous cell carcinoma (Zhang et al., 2020) and esophageal cancer (Wang et al., 2020). The upregulation of circ-ABCB10 was found to promote cell proliferation in various tumors, including esophageal squamous cell carcinoma (Zhang et al., 2020), esophageal cancer (Wang et al., 2020), glioma (Sun et al., 2020), non–small cell lung cancer (Tian et al., 2019), and oral squamous cell carcinoma (Chen et al., 2020). However, the overexpression of circ-ABCB10 was also found to inhibit the migration and invasion of lung cancer cells (Hu et al., 2020; Wu et al., 2020), hepatocellular carcinoma cells (Yang et al., 2020b), and rectal cancer cells (Xian et al., 2020). Generally speaking, these studies indicate that the expression of Circ-ABCB10 is dynamically regulated in tumor progression. The details of its diverse roles are summarized in Table 1.

1) In Proliferation and Invasion

**TABLE 1:** Expression and functions of circ-ABCB10 in different cancers.

Cancer type	Level	Biological function	Related miRNA genes	Target axis	References
Esophageal squamous cell carcinoma (ESCC)	Up	Promote proliferation, migration, and invasion	miR-670-3p	Circ-ABCB10—miR-670-3p	Zhang et al. (2020)
Esophageal cancer (EC)	Up	Promote proliferation, migration, and invasion	miR-203 and slug	Circ-ABCB10— miR-203—slug	Wang et al. (2020)
Glioma (GL)	Up	Promote migration, invasion, and cell cycle progression and inhibits apoptosis	miR-620/FABP5	Circ-ABCB10-miR620/FABP5	Sun et al. (2020)
Non-small-cell lung cancer (NSCLC)	Up	Promote proliferation and migration	miR-1252 and FOXR2	Circ-ABCB10—miR-1252—FOXR2	Tian et al. (2019)
			miR-584-5p, E2F5	Circ-ABCB10—miR-584-5p— E2F5	
Oral squamous cell carcinoma (OSCC)	Up	Promote cell growth and migration	miR-145-5p	Circ-ABCB10— miR-145-5p	Chen et al. (2020)
Epithelial ovarian cancer (EOC)	Up	Promote proliferation, migration, and invasion and reduce apoptosis	miR-1271, Capn4/Wnt/β-catenin miR-1252, miR-203	Circ-ABCB10—miR-1271— Capn4/Wnt/β-catenin	Chen et al. (2019); Lin et al. (2021)
				Circ-ABCB10—miR-1252/miR-203	
Breast cancer (BC)	Up	Promote proliferation, migration, and invasion	miR-223-3p/PFN2 miR-1271	Circ-ABCB10—miR-223-3p—PFN2	Liang et al. (2017); Zhao et al. (2020)
		Enhance glycolysis and colony formation		Circ-ABCB10— miR-1271	
		Mediate PTX resistance, apoptosis, invasion, and autophagy		Circ-ABCB10—Let-7a-5p/DUSP7	
Clear cell renal cell carcinoma (ccRCC)	Up	Promote proliferation and progression. Inhibit apoptosis	miR-331-3p/miR-1228-5p	Circ-ABCB10—miR-331-3p/miR-1228-5p	Huang et al. (2019)
Nasopharyngeal carcinoma (NPC)	Up	Promote growth and metastasis	Rock1	Circ-ABCB10— Rock1	Duan et al. (2020)
Osteosarcoma	Up	Promote proliferation and invasion inhibit apoptosis	miR-203, Bmi-1	Circ-ABCB10—miR-203—Bmi-1	Zhou et al. (2018)
Lung cancer (LC)	Down	Inhibit proliferation and migration	miR-556-3p/AK4	Circ-ABCB10—miR-556-3p—AK4	Hu et al. (2020); Wu et al. (2020)
	Up	Promote the proliferation and migration	miR-217	Circ-ABCB10— miR-217	
Hepatocellular carcinoma (HCC)	Down	Enhance apoptosis	miR-340-5p/miR-452-5P, NRP1, ABL2	Circ-ABCB10—miR-340-5p/miR-452-5P— NRP1/ABL2	Yang et al. (2020b)
		Inhibit proliferation			
		Suppress invasion and migration			
Rectal cancer (RC)	Down	Inhibit ferroptosis and apoptosis	miR-326/CCL5	CircA-BCB10—miR-326—CCL5	Xian et al. (2020)
Cervical cancer (cc)	Down	Inhibits proliferation, invasion, and EMT and promotes apoptosis	miR-128-3p/ZEB1 axis	Circ-ABCB10—miR-128-3p/ZEB1 axis	Feng et al. (2021)

It has been reported that circ-ABCB10 participates in tumor proliferation and migration by sponging several miRNAs *via* various transmitting pathways. It has been found that circ-ABCB10 can sponge miR-1252 and miR-584-5p in non–small cell lung cancer cells and accordingly stimulate the expression of the downstream targeted genes FOXR2 and E2F5 (He et al., 2018). It is worth saying that in the previous study, miR-584-5p exerts a promoting effect on gastric cancer. Thus, we can speculate that circ-ABCB10 may sponge different miRNAs to regulate the same tumor growth. At the same time, circ-ABCB10 can sponge miR-620 and upregulate the expression of the FABP5 axis to promote tumor growth and proliferation in glioma (Sun et al., 2020). In addition, circ-ABCB10 can sponge miR-203 and promote the proliferation of EC cells (Wang et al., 2020). MiR-203 can suppress slug/E-cadherin signals to inhibit cell invasion (Gao et al., 2017a). In addition, circ-ABCB10 can also promote the function of Bmi-1 by sponging miR-203 and enhance the tumor growth in osteosarcoma (Zhou et al., 2018). It indicates that there may be a correlation between circ-ABCB10 and miR-203 in the proliferation of tumor. Furthermore, the overexpression of circ-ABCB10 can stimulate tumor proliferation and invasion by sponging miR-1271, which is also a pivotal miRNA in most regulation processes of circRNAs in breast cancer (Wang et al., 2018) and promote the proliferation and progression of clear cell renal cell carcinoma by activating the target gene—miR-331-3p/miR-1228-5p (Huang et al., 2019). The interesting thing is that in rectal cancer, the number of circ-ABCB10 is decreasing but has an effect of inhibiting ferroptosis and apoptosis by regulating miR-326/CCL5 (Xian et al., 2020). This illustrates that the level of circ-ABCB10 can also have an influence on the proliferation and invasion of tumor. However, in hepatocellular carcinoma, *in vivo* experiments indicate that circ-ABCB10 may be an anti-oncogenic factor (Gao et al., 2017a). With the upregulation of circ-ABCB10, the downstream targeted genes of the signaling axis, namely, NRP1 and ABL2 are upregulated. In this case, it can easily be supposed that circ-ABCB10 can regulate the expression of multiple downstream genes, which has a synergistic effect on tumor growth. Coincidentally, circ-ABCB10 can also regulate the proliferation and EMT of cervical cancer by inhibiting the miR-128-3p/ZEB1 axis (Feng et al., 2021). In general, circ-ABCB10 can act as an oncogene by promoting tumor proliferation and migration in glioma, non–small cell lung cancer, esophageal cancer, breast cancer, and renal cancer (Tang et al., 2017; Deng et al., 2018; Wang et al., 2018; Wang et al., 2020; Zhao et al., 2020) and can also be an anti-oncogene by inhibiting the growth in hepatocellular carcinoma and cervical cancer (Gao et al., 2017a; Feng et al., 2021). It was also shown that circ-ABCB10 plays an obvious role in the proliferation and migration of tumors, with different effects on different cancers.

2) In Metastasis

Metastasis is an important step in cancer progression, and circ-ABCB10 can influence cancer metastasis in different ways (Gao et al., 2017b; Liang et al., 2017; Tang et al., 2017; Zhao et al., 2021). In oral squamous cell carcinoma, circ-ABCB10 was found to be upregulated and related to metastasis and tumor clinical staging of oral squamous cell carcinoma (OSCC) patients, aggravating the progression of OSCC by sponging miR-145-5p (Chen et al., 2020). In breast cancer, circ-ABCB10 acts as a miR-223-3p sponge and regulates the expression of the miR-223-3p targeted gene PFN2 to induce cell migration (Zhao et al., 2021). In addition, it can also promote invasion by sponging miR-1271 and reducing IR sensitivity (Tang et al., 2017). Similarly, the expression of ROCK1 is upregulated and is correlated with circ-ABCB10 expression in NPC cells (Liang et al., 2017). ROCK1 was upregulated following the overexpression of circ-ABCB10 in NPC (Liang et al., 2017). It is demonstrated that circ-ABCB10 has a positive role in cancer metastasis. Similar to proliferation and invasion, circ-ABCB10 also has an adverse effect on tumor metastasis. It is also seen in hepatocellular carcinoma that circ-ABCB10 can suppress the migration and metastasis by inhibiting the miR-340-5p/miR-452-5P—NRP1/ABL2 axis (Yang et al., 2020b). Thus, circ-ABCB10 plays a key role in tumor metastasis in different cancers.

3) In Angiogenesis

Angiogenesis, the creation of new blood vessels, plays a significant role in bone regeneration and osteoblast differentiation and provides essential nutrients and oxygen during bone formation (Hu et al., 2013; Kusumbe et al., 2014). Recently, it was found that circRNAs may be involved in the progression of angiogenesis during the occurrence and development of disease (Zheng et al., 2016; Zhong et al., 2016; Gao et al., 2017b; Deng et al., 2018; He et al., 2018). For example, there is evidence that circ-IARS in pancreatic cancer cells can influence human umbilical vein endothelial cell (HUVEC) monolayers to promote tumor metastasis (Li et al., 2018b). This fact indicates that circ-ABCB10 may also influence the angiogenesis of HUVECs. Vascular endothelial growth factor (VEGF) is another significant cytokine that promotes angiogenesis (Siveen et al., 2017). Kim et al. found that human amnion–derived mesenchymal stem cells (hAMSCs) can promote angiogenesis (Kim et al., 2012), and further research confirmed that hAMSCs can significantly increase the expression of VEGF and circ-ABCB10 (Tang et al., 2020). This implies that circ-ABCB10 may promote cell growth through related pathways. A series of experiments demonstrated that circ-ABCB10 can enhance the angiogenesis of HUVECs by sponging miR-29b-3p and VEGFA (Tang et al., 2020). It was also demonstrated that conditioned medium from hAMSCs (hAMSC-CM) can indirectly promote circ-ABCB10 transcription but not the release of hAMSC-CM by exosomes (Tang et al., 2020). However, there are still numerous questions related to the mechanisms through which circ-ABCB10 may promote angiogenesis.

## Conclusion

In recent years, it has been found that circRNAs originate from the cyclization of pre-mRNAs and have many unique features not found in other RNAs. Because of their unique stable structure and tissue-specific expression, circRNAs have potential applications as novel biomarkers for assessing tumor progression (Jin et al., 2016; Zhang et al., 2017; Jakobi and Dieterich, 2019) and for the diagnosis and treatment of tumors (Lux and Bullinger, 2018; Ouyang et al., 2018). Increasing numbers of studies have found that circRNAs are abnormally expressed during tumorigenesis. In this article, we discussed and reviewed the role of circ-ABCB10 in different cancers and comprehensively summarized a list of related signaling pathways in various tumors. Circ-ABCB10 was found to promote the proliferation, migration, and metastasis of diverse cancers, such as esophageal squamous cell carcinoma, lung cancer, epithelial ovarian cancer, breast cancer, and hepatocellular carcinoma. Moreover, some studies demonstrated that circ-ABCB10 may also contribute to angiogenesis (Tang et al., 2020). From this perspective, circ-ABCB10 might be a potential biomarker for the diagnosis and prognosis of human cancers. However, the knowledge on the roles of circ-ABCB10 in cancer is still limited, and there are still many unresolved problems. For example, there is a lack of knowledge related to the relevant set of miRNA genes for each cancer. In addition, it is also necessary to identify the main sponging targets of circ-ABCB10. Understanding these processes may lead to completely new tumor therapies.
